# Cholestatic Liver Disease and Pregnancy: A Systematic Review and Meta-Analysis

**DOI:** 10.3390/jcm11041068

**Published:** 2022-02-18

**Authors:** Bryan Ferrigno, Romelia Barba, Esli Medina-Morales, Hirsh Trivedi, Vilas Patwardhan, Alan Bonder

**Affiliations:** 1 Department of Medicine, Beth Israel Deaconess Medical Center, Boston, MA 02215, USA; bwferrig@bidmc.harvard.edu; 2 Division of Gastroenterology and Hepatology, Beth Israel Deaconess Medical Center, Boston, MA 02215, USA; rbarbabe@bidmc.harvard.edu (R.B.); jemedina@bidmc.harvard.edu (E.M.-M.); htrived1@bidmc.harvard.edu (H.T.); vpatward@bidmc.harvard.edu (V.P.)

**Keywords:** cholestatic liver diseases, primary biliary cholangitis, primary sclerosing cholangitis, pregnancy, maternal complications, fetal complications

## Abstract

Primary biliary cholangitis (PBC) and primary sclerosing cholangitis (PSC) are two types of chronic cholestatic liver disease (CCLD). Little is known regarding the relationship between these conditions and pregnancy. We performed a systematic review and meta-analysis regarding the maternal and fetal outcomes amongst patients with a known diagnosis of PBC and PSC undergoing pregnancy. Our analysis shows that patients with PBC and PSC who undergo pregnancy are at an increased risk of pre-term delivery, as well as the development of new or worsening pruritus during pregnancy. Additionally, patients with PBC are at higher risk of undergoing a biochemical disease flare during the postpartum period compared to during pregnancy. However, there were no documented cases of maternal mortality or development of decompensated cirrhosis during pregnancy or the postpartum period.

## 1. Introduction

Primary biliary cholangitis (PBC) and primary sclerosing cholangitis (PSC) are two types of autoimmune chronic cholestatic liver disease (CCLD). PBC is characterized by T-cell-mediated destruction of intralobular bile ducts, whereas PSC is characterized by fibrosis and destruction of both intra- and extra- lobular bile ducts [1,2]. PBC primary affects woman, typically those in their fourth and fifth decade of life, whereas PSC is more common in men, with a median age of diagnosis of 40 years old. Both diseases are relatively rare, with PBC diagnosed in 19–402 cases per million persons, and PSC diagnosed in 56 per million persons [3,4,5]. Both conditions carry an increased risk of portal hypertension, cirrhosis, and its related complications.

Given the relatively low prevalence of PBC and PSC, the male predominance seen in PSC, and the average age at diagnosis, particularly in PBC, there is a scarcity of studies describing either the effect of these conditions on pregnancy or the effect of pregnancy on these conditions. Some studies suggested an increased risk of pre-term birth, gestational complications, and development of a disease flare as a result of pregnancy in patients with CCLD [6,7,8]. However, these associations are inconsistently reported, and there are conflicting data among the limited studies that have analyzed these outcomes [9,10]. 

Based on observational studies, we hypothesized an increase in fetal and maternal complications in patients with PBC and PSC who undergo pregnancy, and that there may be a correlation between pregnancy and the risk of developing a disease flare, as defined by liver biochemical tests that are outside of pre-defined parameters [8,11,12]. The objective of this study was to perform a systematic review and meta-analysis to evaluate the association of PBC and PSC with maternal and fetal outcomes in pregnancy.

## 2. Methods

Our systematic review and meta-analysis was based on the requirements of the Preferred Reporting Items for Systematic Review and Meta-analysis (PRISMA) Statement [13]. The protocol was submitted to the International Prospective Register of Systematic Reviews (PROSPERO), registration ID 242285.

### 2.1. Search Strategy

We performed a systematic literature search of articles indexed in PubMed, EMBASE, Web of Science, and Cochrane Library for studies published through September 2021. The search strategy was based on recommendations from the Cochrane Handbook for Systematic Reviews and reported according to the guidelines of meta-analysis of observational studies in epidemiology [14]. The search terms included “Liver cirrhosis, Biliary” OR “Cholangitis, Sclerosing” AND “Pregnancy” OR “Pregnant women” OR “Gravidity”. The complete details of the search strategy and subject terms can be found in Table S1.

### 2.2. Study Inclusion Criteria

We included studies that met the following inclusion criteria: (1) Confirmed diagnosis of Primary Biliary Cholangitis (PBC) or Primary Sclerosing Cholangitis (PSC) as defined by the American Association for the Study of Liver Diseases (AASLD) or the European Association for the Study of the Liver (EASL) with a diagnosis made either before, during, or within six months from delivery or pregnancy loss (postpartum period); (2) Females with at least one pregnancy; (3) Study designs that consisted of case-control studies, cohort studies (prospective or retrospective), cross-sectional studies, and case series; (4) Full text articles available in the English language. Grey literature, such as conference abstracts, was also accepted for analysis. Diagnostic criteria for the diagnosis of PBC and PSC per AASLD and EASL guidelines are similar and are further described in Supplementary Table S2 [15,16,17,18,19].

### 2.3. Study Exclusion Criteria

We excluded female patients with a diagnosis of PBC or PSC made after 6 months from delivery or pregnancy loss. Additionally, review articles, letters, commentaries, studies with a pediatric population, case reports that included less than three patients, patients with autoimmune hepatitis (AIH)/PBC or AIH/PSC overlap syndromes, grey literature that did not reveal data on outcomes of pregnancy, patients with a history of a prior liver transplant, animal-based studies, and studies in languages other than English were excluded. If multiple articles were published by the same group using similar cohorts, we selected either the more recent or the higher quality publication.

The studies retrieved from the database search were evaluated against eligibility for inclusion using the Covidence software (Covidence systematic review software, Veritas Health Innovation, Melbourne, Australia. Available at www.covidence.org) by two investigators (R.B.B. and B.F.) independently [20]. Excluded studies with the reason for exclusion are available in Table S3. In the case of discrepancies in the inclusion of a study, the agreement was reached by consensus and/or by consulting a third investigator (E.M.-M.).

### 2.4. Quality Assessment of Included Studies

Quality assessment of the included cohort, and case-control studies was performed using the Newcastle-Ottawa Scale (NOS) and the case series studies were assessed using The Canada Institute of Health Economics (IHE) Quality Appraisal Tool for Case Series (Tables S4–S6) [21,22]. Two reviewers assessed the quality of eligible studies independently. The NOS is a standard quality assessment tool used to evaluate the quality of the observational study on the basis of three domains: recruitment and selection of the participants, similarity, and comparability between the groups, and ascertainment of the outcome of interest among case-control studies. A study that scored above 6 points was considered as good quality. 

The IHE checklist was tailored to our project with a total of 17 criteria distributed in seven domains (complete list on Table S6), we gave one point for each “yes” question and by consensus, and a score above 14 points was considered good quality. Based on the score achieved by the individual study, a good, fair, or poor quality of the study was determined. Agreement regarding the marking of each study was reached by consensus. Finally, the Grading of Recommendations, Assessment, Development and Evaluation (GRADE) methodology was used to assess the quality of evidence of each study. Two authors (R.B.B. and B.F.) independently assessed the risk of bias, inconsistency, imprecision, and publication bias. The overall quality was graded using the GRADEPro Guideline Development tool [23]. 

### 2.5. Outcomes 

We analyzed both maternal and fetal outcomes. Primary outcomes include the rates of maternal and fetal mortality, preterm birth, and the development of a biochemical disease flare as defined below. 

Secondary outcomes include presence of new or worsening pruritus, the development of gestational complications, including gestational diabetes, gestational hypertension, and Pre-eclampsia, as well as low birth weight, rate of birth defects, and the development of decompensated cirrhosis.

We performed a subgroup analysis amongst patients with PSC assessing if acute cholangitis was more likely to occur during pregnancy versus the postpartum period. 

### 2.6. Definitions

A biochemical disease flare in PBC patients was defined as, according to prior studies by Efe et al, and Trivedi et al, an alkaline phosphatase greater than 3× the upper limit of normal, aspartate transaminase (AST) greater than 2× the upper limit of normal, or total bilirubin greater than 1× the upper limit of normal [8,11]. In addition, patients’ pre-pregnancy biochemical values were considered if available, and only abnormal values higher than pre-pregnancy values were counted towards qualifying as a flare. 

A biochemical flare in PSC patients was defined as a rise in the alkaline phosphatase, AST, ALT, or total bilirubin greater than two times their pre-pregnancy value. When pre-pregnancy biochemical data were not available, a flare was defined as two-time elevations in the markers mentioned above compared to the upper limit of normal. 

In both groups, an isolated rise in the alkaline phosphatase alone did not qualify as a disease flare, as this is a known phenomenon that can occur during normal pregnancy. 

It should be noted that the definition of disease flare in both conditions is imperfect and may not truly reflect the underlying disease activity, particularly given that other conditions such as intrahepatic cholestasis of pregnancy may be contributing. However, utilizing available biochemical data as a surrogate to underlying disease activity is a reasonable measure.

Decompensated cirrhosis was defined as the development of variceal hemorrhage, ascites, SBP, hepatorenal syndrome, hepatocellular carcinoma, or hepatopulmonary syndrome during pregnancy or postpartum.

Preterm birth was defined according to the American College of Obstetricians and Gynecologists classification system, as delivery of the child before 37 weeks gestation [24]. 

Fetal death was stratified into spontaneous abortion (miscarriage), defined as pregnancy loss before the 20th week of gestation, and intrauterine fetal demise (stillbirth), defined as pregnancy loss after the 20th week of gestation, if the data were available [25,26].

### 2.7. Data Extraction

Quantitative and qualitative data were extracted from each study by two independent investigators (R.B.B. and B.F.) using a customized template created in Covidence™, and disagreements were resolved by a third investigator (E.M-M.). When available, data extracted included study author, year of publication, country of origin, population demographics, number of patients, number of pregnancies, maternal and fetal outcomes, as described above. Quality assessment of studies was performed using the Quality assessment tool on Covidence adapted for NOS or IHE checklist when suitable.

### 2.8. Statistical Analysis

We used Freeman–Tukey double arcsine transformation to calculate standardized proportions with 95% confidence intervals (CI) [27]. Then, we performed a random effect meta-analysis of proportions, described by Dersimonian and Laird, to calculate the event rates with 95% CI for the maternal and fetal outcomes [27]. We used odds ratio (OR) with 95% intervals with a random effect model to compare between dichotomous variables. Heterogeneity was expressed as I^2^ statistic values using a threshold of 50% to consider significant heterogeneity. We assessed the publications bias using Egger’s regression model. All analyses were performed using STATA 13.1 (College Station, TX, USA, StataCorp LP). 

## 3. Results

### 3.1. Study Research Results

**Figure 1:**
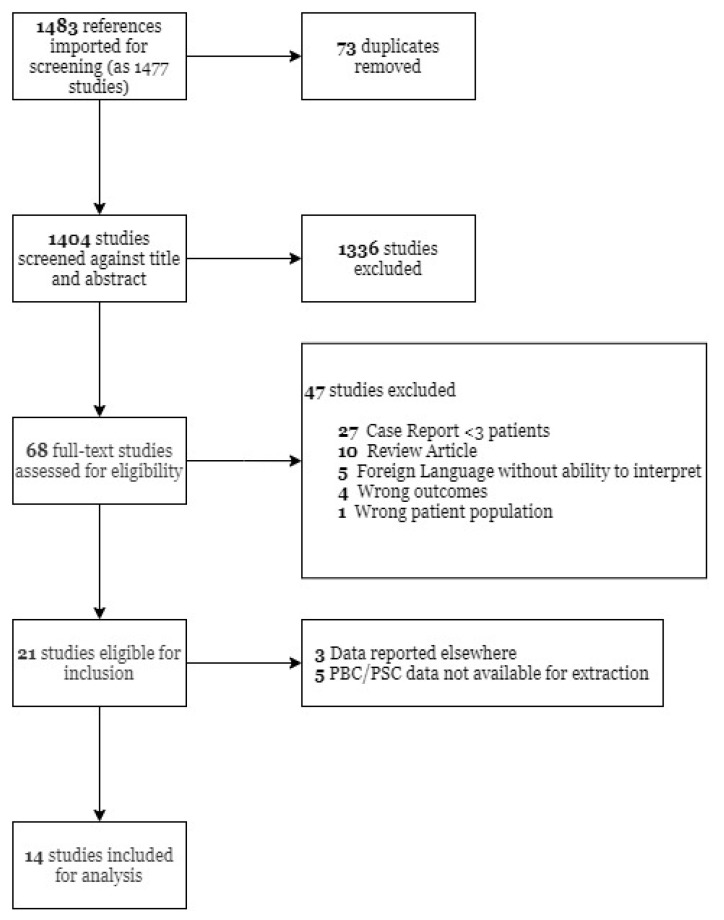
Flow diagram of the studies included in the systematic review and meta-analyses. PBC—Primary biliary cholangitis; PSC—Primary sclerosing cholangitis.

Our initial study search yielded 1483 studies. After abstract screening and full text review, 14 studies were included for analysis. Figure 1 contains the flow diagram for our search.

### 3.2. Description of Included Studies

Of the 14 studies included, we included a total of 119 patients and 219 pregnancies with PBC and 399 patients and 469 pregnancies with PSC. The mean age in patients with PBC was 32.8 ± 5.5 and 31 ± 6 in patients with PSC. Among the included PBC studies, four were case series, two were case controls, and three were cohort. From the studies of PSC, three were case series, three were case-control studies, and one was a cohort study. Included studies were from 1968 to 2020 and 1996 to 2021 for PBC and PSC, respectively. Table 1 and Table 2 display the individual study characteristics for both PBC and PSC studies.

### 3.3. Maternal and Fetal Outcomes

The event rates (ER) for maternal and fetal outcomes for primary biliary cholangitis and primary sclerosing cholangitis are shown in the Table 3 and Table 4, respectively. There were no documented cases of maternal mortality or the development of decompensated cirrhosis amongst available data.

#### 3.3.1. Fetal Outcomes

**Figure 2:**
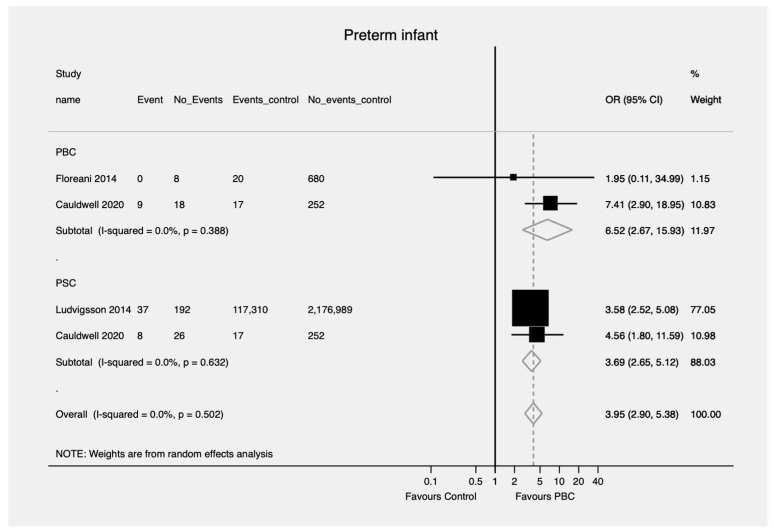
Preterm birth (the event) in patients with PBC and PSC compared to controls. PBC—Primary biliary cholangitis; PSC—Primary sclerosing cholangitis; OR—Odds ratio; CI—Confidence interval.

**Figure 3:**
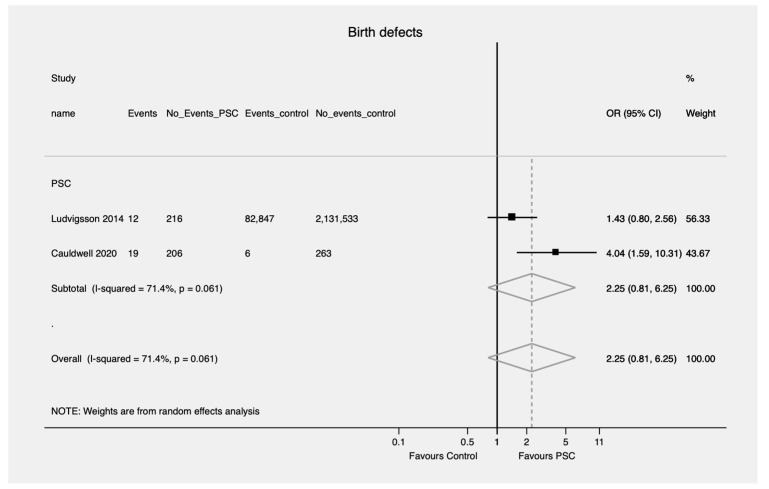
Birth defects (the event) in patients with PSC. PSC—Primary sclerosing cholangitis; OR—Odds ratio; CI—Confidence interval.

Patients with PBC and PSC were more likely to have a preterm birth compared to controls (OR = 6.52; 95% CI, 2.67—15.93 and OR = 3.69; 95% CI, 2.65—5.12, respectively) (Figure 2). No evidence of heterogeneity was found (I^2^ = 0.0, *p* = 0.388 and I^2^ = 0.0, *p* = 0.502, respectively). There was no difference in the likelihood of birth defects in patients with PSC compared to controls (OR = 2.25; 95% CI, 0.81—6.25) (Figure 3). However, there was significant heterogeneity in the findings (I^2^ = 71.4%, *p* = 0.061). No pooled analysis was feasible comparing birth defects in PBC patients compared to controls. 

Regarding fetal mortality, the PBC cohort had a spontaneous abortion (SA) ER of 13% (95% CI 0–34%) and an intra-uterine fetal demise (IUFD) ER of 0% (95% CI 0–3%). The PSC cohort had a SA ER of 2% (95% CI 0–8%) and an IUFD ER of 0% (95% CI 0–3%). There was not sufficient data to perform comparisons between controls regarding fetal mortality in either the PBC or PSC cohort.

#### 3.3.2. Pruritus

**Figure 4:**
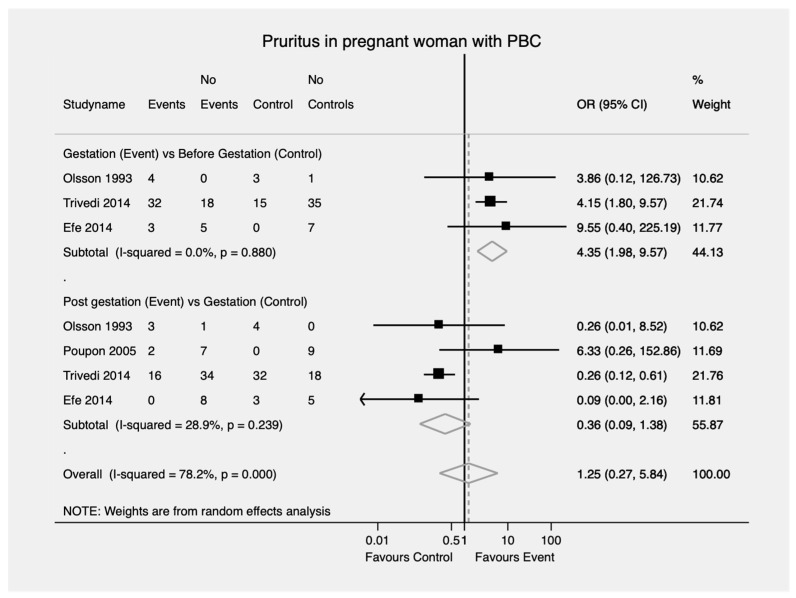
Pruritus in pregnant woman with PBC. PBC—Primary biliary cholangitis; OR—Odds ratio; CI—Confidence interval.

Women with PBC were more likely to develop new or worsening pruritus during gestation compared to prior to the onset of pregnancy (OR = 4.35; 95% CI, 1.98—9.57). No difference was found between the likelihood of developing new or worsening pruritus during gestation compared to the postpartum period (OR = 0.36; 95% CI, 0.09—1.38) (Figure 4). No significant heterogeneity was found in both cases (I^2^ = 0%, *p* = 0.880 and I^2^ = 28.9%, *p* = 0.239, respectively).

**Figure 5:**
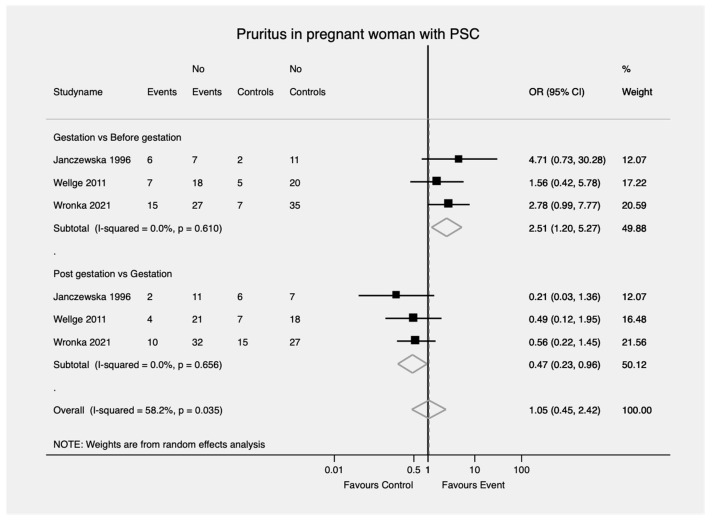
Pruritus in pregnant woman with PSC. PSC—Primary sclerosing cholangitis; OR—Odds ratio; CI—Confidence interval.

Women with PSC were more likely to develop new or worsening pruritus during gestation compared to before the onset of pregnancy as well as compared to the postpartum period (OR = 2.51; 95% CI, 1.20—5.27 and OR = 0.47; 95% CI, 0.23—0.96) (Figure 5). No heterogeneity was found in either comparison (I^2^ = 0%, *p* = 0.61 I^2^ = 54.9%, *p* = 0.05, respectively).

#### 3.3.3. Biochemical Flare Rate

**Figure 6:**
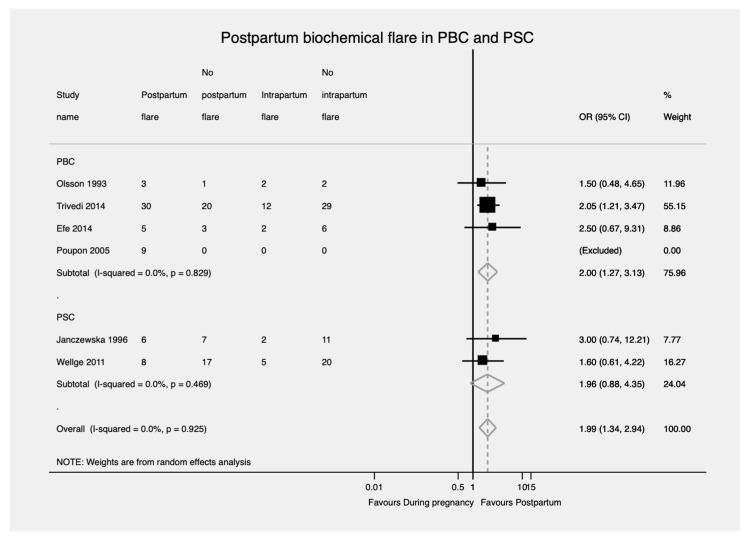
Biochemical flare during postpartum in PBC and PSC. PBC—Primary biliary cholangitis. PSC—Primary sclerosing cholangitis; OR—Odds ratio; CI—Confidence interval.

Patients with PBC were significantly more likely to develop a biochemical flare during the postpartum period compared to during pregnancy, whereas among patients with PSC there was no significant difference between the flare rates during the postpartum period compared to during pregnancy (OR = 2.00; 95% CI, 1.27—3.13 and OR = 1.96; 95% CI, 0.88—4.35, respectively) (Figure 6). No evidence of heterogeneity was found in either case (I^2^ = 0.0%, *p* = 0.829 and I^2^ = 0.0%, *p* = 0.469, respectively). 

#### 3.3.4. Acute Cholangitis in PSC

**Figure 7:**
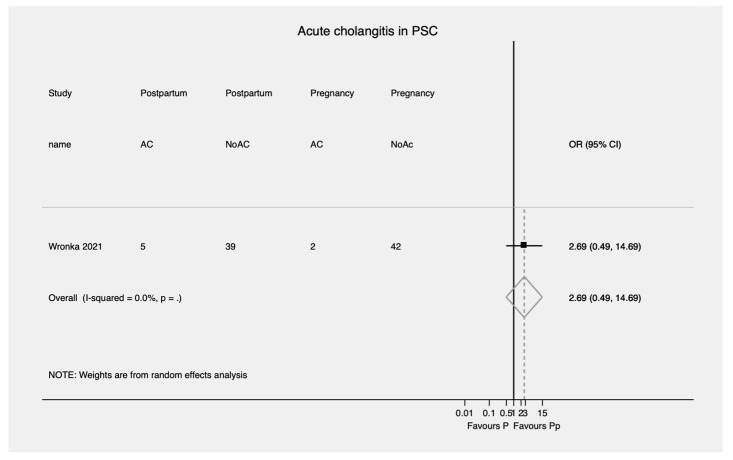
Acute cholangitis during pregnancy and postpartum in PSC. PSC—Primary sclerosing cholangitis; OR—Odds ratio; CI—Confidence interval; AC—Acute cholangitis; Pp—Postpartum; P—Pregnancy.

In patients with PSC, there was no significant difference between the risk of developing acute cholangitis during postpartum compared to during pregnancy (OR = 2.69; 95% CI, 0.49–14.69) (Figure 7).

### 3.4. Quality Assessment of Included Studies and Publication Bias

A quality assessment was conducted on all included studies. Case-control, cohort, and cross-sectional studies were assessed using the Newcastle-Ottawa scale, while case series were assessed using the IHE checklist (Table 5, Table 6 and Table 7). The scores varied. The average score for case-control and cohort studies was good. The cross-sectional score was fair, only including one study with limited information because it was an abstract. For the case series, the average score was fair.

The evidence level for our outcomes of interest was assessed using the GRADE protocol, as mentioned above. The evidence level for preterm birth in both PSC and PBC, as well as birth defects in PSC when compared to controls, was graded as “low.” For new or worsening pruritus during pregnancy when compared to before pregnancy, the evidence was graded as “low” for both PBC and PSC. For new or worsening pruritus during pregnancy compared to the postpartum period, the evidence level was graded “very low” for both PBC and PSC. Lastly, for risk of biochemical flare during the postpartum period compared to flare during pregnancy, the evidence level was graded “very low” for both PSC and PBC (Tables S7–S11).

Due to the limited number of included studies for each condition (<10), we did not perform publication bias analysis.

## 4. Discussion

There is a scarcity of studies describing the outcomes of pregnancy amongst patients with a known diagnosis of PBC or PSC. Studies are typically limited to case reports or small case series, and existing data is conflicting. Some prior reports suggested an increased risk of fetal and maternal complications during pregnancy. However, these findings have not been universal [6,7,8,9,10,34]. As a result, if these patients choose to undergo pregnancy, managing their condition can be a significant challenge given the lack of outcome data to guide clinical decision making and counseling. Additionally, the lack of consistent data impedes the ability to publish consensus guidelines on monitoring and caring for this patient population. 

Our study showed that pregnant women with PBC and PSC are about six and four times more likely to have premature births, respectively, compared to healthy controls. Preterm birth is associated with a significantly increased risk of perinatal mortality and neonatal morbidity [35]. Previous studies have also demonstrated an increased risk of preterm birth among patients with intrahepatic cholestasis of pregnancy. However, it is uncertain whether this data can be extrapolated to patients with PBC and PSC [36]. A potential explanation for this increased risk of premature birth in patients with PBC and PSC may be an increased number of induced pregnancies due to worsening of the symptoms such as pruritus [7]. However, other studies have suggested that patients with autoimmune diseases are at an increased risk of poor fetal outcomes, including preterm delivery [37]. Regardless, physicians caring for this patient population should provide appropriate counseling and guidance regarding the potential increased risk of preterm birth. Additionally, optimization of the patient’s other comorbid conditions is an appropriate strategy to minimize the risk of undergoing preterm birth [38]. 

We analyzed the rates of biochemical disease flare using predefined criteria as previously described by Efe et al. and Trivedi et al. [8,11]. Comparing the rates of disease flare during pregnancy and the postpartum period, patients with PBC were significantly more likely to develop a biochemical disease flare during the postpartum period. No difference in flare rate was found in patients with PSC. It is a well-studied phenomenon that certain autoimmune diseases, such as rheumatoid arthritis and multiple sclerosis, can become quiescent during pregnancy with significant symptomatic improvement [39,40]. This finding, while well studied, is poorly understood. The role of increased expression and activity of regulatory T-cells has been hypothesized to support lower levels of inflammation and overall disease activity during pregnancy [41]. Others theorize that novel human leukocyte antigens (HLA) from the fetus that are circulating in the maternal bloodstream induce temporary changes in the maternal immune system, resulting in improved tolerance of “self” antigens [39]. Given the pathophysiology of PBC is thought to be largely autoimmune in nature, adapting this same principle is one potential explanation as to why flare rates are lower during the pregnancy. After birth, there is a subsequent return to pre-pregnancy immunologic activity during the postpartum period, resulting in higher rates of biochemical disease flare. Physicians caring for these patients should be aware that postpartum flares are more likely to occur and could consider changing management if biochemical tests do not return to baseline after several months.

With regards to pruritus, in both patients with PBC and PSC, we found a statistically significant higher rate of developing new or worsening of pruritus during pregnancy. Pruritus can be debilitating, and if unmanageable can result in early induction of the pregnancy as mentioned above. Interestingly, as mentioned, while pruritus was a predominant feature during pregnancy, occurring in 25% of pregnant patients with PBC and 14% of pregnant patients with PSC, the rates of biochemical flares in both conditions were not higher during gestation. Amongst PBC patients with available information, 54% were on UDCA during pregnancy, but this alone did not appear to be effective at treating or preventing pruritus. Patients should be informed of the increased likelihood of pruritus during pregnancy, and aggressive symptomatic therapy should be considered in patients with refractory symptoms.

Lastly, it should be noted that among included studies across both the PBC and PSC groups, there were no documented cases of maternal mortality during pregnancy or the immediate postpartum period, nor were there any cases of development of decompensated cirrhosis. Additionally, among PBC and PSC patients who underwent pregnancy, there was no associated increased risk of having a child with birth defects.

Our study has several strengths. Our study is the first of its nature to pool and analyze all available data on pregnancy-related outcomes in patients with previously known PBC and PSC. Second, the maternal and fetal outcomes analyzed in our review can serve as valuable information for physicians caring for this population, and can assist with clinical decision making, counseling, and expectant management. Additionally, by setting strict inclusion and exclusion criteria regarding the timeline of diagnosis of PBC and PSC in relation to pregnancy, our outcome data have a high likelihood of accurately analyzing the impact of these conditions on pregnancy.

There are several limitations to our systematic review. The first and perhaps largest limitation is the scarcity of available studies. Due to the relative rarity of these conditions, studies available analyzing pregnancy outcomes were quite limited. Second, among available studies, only a minority of them had controls available. Third, sufficient data were not available to stratify outcomes by presence of cirrhosis, portal hypertension, cirrhosis stage, or those taking UDCA, which would have provided useful information for physician counselling and expectation management. Finally, it should be noted that for several of the outcomes analyzed, a particularly large sample size in one study (Ludvigsson et al.) had a predominant impact on the calculations compared to other studies. Many of our included studies had relatively small sample sizes, and individually they are likely not powerful enough to accurately assess the outcomes of interest.

Future multi-centered studies with larger sample sizes with appropriate controls are warranted to further validate the findings of our meta-analysis.

## 5. Conclusions

Our study has shown that pregnancy appears to be relatively safe for mothers with PBC and PSC. There were no documented cases of maternal mortality during pregnancy or the post-partum period. However, there is an associated increase in the risk of preterm birth in both conditions, as well as an increased likelihood of postpartum disease flare in patients with PBC. In addition, we found an increased likelihood of developing new or worsening pruritus during pregnancy in both conditions, but particularly in PSC. These findings serve as valuable information for physicians caring for this patient population, and can help guide clinical decision making, counseling, and expectant management.

## Figures and Tables

**Table 1:** Individual study characteristics for primary biliary cholangitis studies.

Study (Year)	Study Design	Country	Total Participants (Pregnancies)	Primary Outcome	Maternal Outcomes	Fetal Outcomes	Concurrent IBD	Biochemical/Immunological Parameters	Pregnancy Biochemical Exacerbation	Postpartum Biochemical Exacerbation
Whelton 1968 [28]	Case series	United Kingdom	5 (6)	To investigate pregnancy outcomes in women with PBC and the influence of pregnancy on the course of their disease.	Jaundice Pruritus Toxaemia Maternal mortality	Mode of delivery Pre-term delivery Miscarriage Stillbirth Neonatal death	NR	Bilirubin	Unclear	Unclear
Olsson 1993 [29]	Case series	Sweden	3 (4)	To report data from pregnancies in PBC patients	Jaundice occurrence Biochemical profile during pregnancy Pruritus	Mode of delivery Gestational age Miscarriage Fetal demise	NR	ALP Aminotransferases Bilirubin	Yes	Yes
Poupon 2005 [30]	Case series	France	6 (9)	To report the experience of pregnancies in UDCA-treated PBC patients.	Pruritus Biochemical profile during pregnancy	Birth weight Mode of delivery Miscarriages Fetal complications	NR	ALP, ALT Serum bile acids Bilirubin Immunoglobulin G and M Antimitochondrial antibodies	Yes	Yes
Kumagi 2009 [31]	Retrospective cohort	Canada	33 (107)	To delineate the clinical characteristics associated with pregnancy and PBC.	Pruritus Right upper quadrant pain Biochemical flares Cirrhosis/portal hypertension	Stillbirth Miscarriage	NR	NR	Unclear	Unclear
Floreani 2015 [9]	Case-control	Italy	7 (8)	To analyze fertility in PBC and investigate the outcome of pregnancy in women with PBC.	Symptom profile during pregnancy Pruritus	Miscarriages Mode of delivery	NR	ALP, ALT, AST, GGT	NR	Yes
Trivedi 2014 [11]	Retrospective cohort	Canada	32 (50)	To describe data on pregnancy, fetal, and maternal-related outcomes from PBC patients.	Pruritus Disease activity Hepatic decompensation	Miscarriage Stillbirth Elective abortion Birth defects Premature delivery Perinatal death Severe disability	NR	Bilirubin ALP, ALT, AST.	Yes	Yes
Efe 2014 [8]	Case series	Turkey	6 (8)	To report experiences of PBC patients who had pregnancies while on UDCA treatment.	Gestational hypertension, gestational diabetes mellitus, pre-eclampsia Biochemical flare Pruritus Biochemical profile during pregnancy	Mode of delivery Stillbirth Miscarriage IUFD Pre-term labor Congenital malformations Small for gestational age	NR	Bilirubin ALP, AST Immunoglobulin G and M Antimitochondrial antibody	Yes	Yes
Cauldwell 2020 [6]	Retrospective cohort	United Kingdom	27 (27)	To report pregnancy outcomes in women with PBC and PSC.	Gestational diabetes mellitus Pre-eclampsia Gestational hypertension Postpartum hemorrhage	Mode of delivery IUFD Pre-term birth Birth weight	NR	ALT, GGT Serum bile acids Platelets	Unclear	Unclear

PBC—Primary biliary cholangitis; IBD—Inflammatory bowel disease; NR—not reported; UDCA—Ursodeoxycholic acid; PSC—Primary sclerosing cholangitis; ALP—Alkaline phosphatase; ALT—Alanine aminotransferase; ALT—Aspartate aminotransferase; GGT—Gamma-glutamyl transferase; IUFD—Intrauterine fetal death.

**Table 2:** Individual study characteristics for primary sclerosing cholangitis studies.

Study (Year)	Study Design	Country	Total Participants (Pregnancies)	Primary Outcome	Maternal Outcomes	Fetal Outcomes	Concurrent IBD	Biochemical Parameters	Pregnancy Biochemical Exacerbation	Postpartum Biochemical Exacerbation
Janczewska 1996 [12]	Case series	Sweden	10 (13)	To study the pregnancy outcomes in PSC patients and, conversely, the effect of pregnancy on the disease.	Pruritus Abdominal pain Fever Jaundice Biochemical flare	Gestational age Mode of delivery Pre-term delivery APGAR score Birth weight	Yes	Total bilirubin Albumin ALP, AST, ALT	Yes	Yes
Wellge 2011 [10]	Case series	Germany	17 (25)	To study PSC activity during pregnancy and after delivery and the influence of medication on the fetal and maternal outcome.	Maternal death Biochemical disease activity IBD flare Pruritus	Miscarriage Pre-term delivery Perinatal death Severe disability Apgar score Cesarean section	Yes	Bilirubin ALP, ALT GGT	Yes	Yes
Antoniazzi 2011 [32]	Case-control	Italy	10 (17)	To evaluate pregnancy outcomes in PSC patients and the effect of pregnancy on the disease.	Pruritus Transaminase increment IBD exacerbation Cesarean section	Miscarriage Birth weight Apgar index of the first and fifth minutes	Yes	Transaminases Total bile salts	Yes	NR
Ludvigsson 2014 [7]	Case-control	Sweden	229 (229)	To examine pregnancy outcomes among women with PSC.	Spontaneous and induced pre-term birth. Pre-eclampsia Gestational diabetes mellitus	Mode of delivery IUFD Pre-term birth fetal growth Congenital abnormalities	Yes	NR	NR	NR
Patel 2019 [33]	Case-control	United States	64 (109)	To evaluate maternal-fetal outcomes among women with PSC with and without concurrent IBD.	Pruritus Abdominal pain Gestational diabetes Pre-eclampsia	Spontaneous abortions Therapeutic abortions Mode of delivery IUFD Pre-term delivery Birth defects	Yes	NR	NR	NR
Cauldwell 2020 [6]	Retrospective cohort	United Kingdom	34 (34)	To report pregnancy outcomes in women with PBC and PSC.	Gestational diabetes mellitus Pre-eclampsia Gestational hypertension Postpartum hemorrhage	Mode of delivery IUFD Pre-term birth Birth weight	Unclear	ALT, GGT Serum bile acids Platelets	Unclear	Unclear
Wronka 2021 [34]	Case series	Poland	25 (42)	To investigate the outcomes of pregnancy in patients with PSC and the influence of pregnancy on disease course.	Death or liver transplantation during one year after Delivery Pruritus Cholangitis IBD flare	Live births Gestational age Apgar score Birth weight Mode of delivery Pre-term delivery Miscarriages Stillbirth Birth defects	Yes	AST, ALT, GGTP, ALP INR Bilirubin Albumin Platelets	NR	NR

PSC—Primary sclerosing cholangitis; IBD—Inflammatory bowel disease; NR—not reported; PBC—Primary biliary cholangitis; IUFD—Intrauterine fetal death; ALP—Alkaline phosphatase; ALT—Alanine aminotransferase; ALT—Aspartate aminotransferase; GGT—Gamma-glutamyl transferase; INR—International Normalized Ratio.

**Table 3:** Prevalence data of maternal and fetal outcomes in patients with primary biliary cholangitis.

Outcomes	Unit	Number of Cases	Total Number of Patients	Number of Studies	ER (95% CI; Heterogeneity)	Control Group Cases/Total Control	ER (95% CI; Heterogeneity)
Maternal outcomes							
Pregnancy flare	P	16	53	*n =* 3	29% (16–43%)	NA	NA
Postpartum flare	P	47	71	*n =* 4	77% (50–0.96%)	NA	NA
Decompensated cirrhosis	P	0	70	*n =* 4	0% (0–10%)	NA	NA
Pruritus onset during pregnancy	P	41	130	*n =* 8	25% (8–46%)	NA	NA
Gestational hypertension	P	8	31	*n =* 2	23% (9–41%)	NA	NA
Gestational diabetes	P	1	26	*n =* 1	4% (1–19%)	NA	NA
C-Section	P	8	37	*n =* 3	19% (5–38%)	83/351	24 (28–100%)
Postpartum hemorrhage	P	23	43	*n =* 3	53% (2–100%)	NA	NA
Fetal outcomes							
Preterm infant	P	15	97	*n =* 5	16% (1–41%)	37/969	4 (3–5%)
SA	P	61	202	*n =* 8	13% (0–34%)	159/969	15 (13–17%)
IUFD	P	5	202	*n =* 8	0% (0–3%)	NA	NA
Birth defects	P	1	50	*n =* 1	2% (0–10%)	NA	NA
Low birth weight	P	3	43	*n =* 3	5% (0–16%)	NA	NA

P—Per pregnancy; ER—Event rate; CI—Confidence intervals; NA—Not available; SA—Spontaneous abortion; IUFD—Intra-uterine fetal demise.

**Table 4:** Prevalence data of maternal and fetal outcomes in patients with primary sclerosing cholangitis.

Outcomes	Unit	Number of Cases	Total number of Patients	Number of Studies	ER (95% CI; Heterogeneity)	Control Group Cases/Total Control	ER (95% CI; Heterogeneity)
Maternal outcomes							
Pregnancy flare	P	8	55	*n =* 3	14% (5–25%)	NA	NA
Postpartum flare	P	14	38	*n =* 2	37% (22–53%)	NA	NA
Pruritus onset during pregnancy	P	6	38	*n =* 2	14% (4–28%)	NA	NA
Gestational hypertension	P	4	34	*n =* 1	12% (5–27%)	NA	NA
Gestational diabetes	P	2	263	*n =* 2	0% (0–2%)	NA	NA
Preeclampsia	P	8	263	*n =* 2	3% (1–5%)	NA	NA
C-section	P	100	312	*n =* 5	34% (25–43%)	NA	NA
Postpartum hemorrhage	P	30	81	*n =* 3	26% (3–58%)	NA	NA
IBD flare	P	6	42	*n =* 4	13% (3–26%)	NA	NA
AC during pregnancy	P	13	178	*n =* 3	7% (3–11%)	NA	NA
AC during postpartum	P	6	7	*n =* 2	10% (3–20%)	NA	NA
Fetal outcomes							
Preterm infant	P	87	410	*n =* 5	19% (9–30%)	117,327/2,294,568	5% (5–5%)
SA	P	6	326	*n =* 5	2% (0–8%)	NA	NA
IUFD	P	9	106	*n =* 4	5% (0–18%)	60,027/2,304,863	3 (3–3%)
Birth defects	P	18	350	*n =* 3	3% (2–5%)	82,853/2,214,649	4% (4–4%)
Low birth weight	P	48	544	*n =* 6	8% (5–10%)	NA	NA

P—Per pregnancy; ER—Event rate; CI—Confidence intervals; NA—Not available; IBD—Inflammatory bowel disease; SA—Spontaneous abortion IUFD—Intra-uterine fetal demise; AC—Acute cholangitis.

**Table 5:** Quality assessment of individual case-control studies using the Newcastle-Ottawa Scale.

Case-Control	Selection				Comparability	Outcome			
Study	Is the Case Definition Adequate	Representativeness of the Cases	Selection of Controls	Definition of Controls	Comparability of Cases and Controls on the Basis of Design or the Analysis	Ascertainment of Exposure	Same Method of Ascertainment for Cases and Controls	Non-Response Rate	Overall Score
Antoniazzi 2011	✓	✓	✓	✕	✓✓	✓	✕	✕	Fair (6)
Floreani 2014	✓	✓	✓	✓	✓✓	✓	✓	✕	Good (8)
Ludvigsson 2014	✓	✓	✓	✓	✓✓	✓	✓	✓	Good (8)
Patel 2019	✓	✓	✕	✕	✓	✓	✓	✕	Fair (5)

✓—Yes; ✕—No.

**Table 6:** Quality assessment of individual cohort studies using the Newcastle-Ottawa Scale.

Cohort	Selection				Comparability	Outcome			
Study	Representativeness of the Exposed Cohort	Selection of the Non-Exposed Cohort	Ascertainment of Exposure	Demonstration that Outcome of Interest was not Present at Start of Study	Comparability of Cohorts on the Basis of the Design or Analysis	Assessment of Outcome	Was Follow-up Long Enough for Outcomes to Occur	Adequacy of Follow-up of Cohorts	Overall Score
Trivedi 2014	✓	✓	✓	✕	✓	✓	✓	✓	Good (7)
Kumagi 2009	✓	✓	✓	✕	✓	✓	✓	✓	Good (7)
Cauldwell 2020	✓	✓	✓	✓	✓	✓	✓	✓	Good (8)

✓—Yes; ✕—No.

**Table 7:** Quality assessment of individual case series studies using the IHE Quality Appraisal Tool.

Study	Study Objective	Study Design	Study Population	Outcome Measures	Statistical Analysis	Results and Conclusions	Competing Interests and Sources of Support	Overall Score
Whelton 1968	✓	✓	✓	✓✓✓✓	Partial	✓✓✓	✕	Fair (10)
Olsson 1993	✓	✓	✓✓	✓✓	Partial	✓✓✓	✕	Poor (9)
Janczewska 1996	✓	✓	✓✓	✓✓✓✓	Partial	✓✓✓	✕	Good (14)
Poupon 2005	✓	✓	✓✓	✓✓✓✓	Partial	✓✓✓✓	✕	Fair (12)
Wellge 2011	✓	✓✓	✓✓✓	✓✓✓✓	✓	✓✓✓✓	✓	Good (16)
Efe 2014	✓	✓	✓✓	✓✓✓✓	✓	✓✓✓	✓	Fair (13)
Wronka 2021	✓	✓✓	✓✓✓	✓✓✓✓	✓	✓✓✓✓	✓	Good (16)

✓—Yes; ✕—No. IHE—Institute of Health Economics.

## Data Availability

All data used for the analyses is available within the manuscript and the original publications of the included studies.

## Supplementary Materials

The following are available online at https://www.mdpi.com/article/10.3390/jcm11041068/s1, Table S1: Search strategy, Table S2: Diagnostic criteria of Primary Biliary cholangitis and Primary Sclerosing Cholangitis based on AASL and EASL guidelines, Table S3: Excluded studies, Table S4: Assessment of quality of a Cohort study—Newcastle Ottawa Scale, Table S5: Assessment of quality of a Case-Control study—Newcastle Ottawa Scale, Table S6: Quality Appraisal Checklist for Case Series Studies, Table S7: GRADE assessment for fetal outcomes in PSC pregnancies, Table S8: GRADE assessment for fetal outcomes in PBC pregnancies, Table S9: GRADE assessment for pruritus in pregnant woman with PBC, Table S10: GRADE assessment for pruritus in pregnant woman with PSC, Table S11: GRADE assessment for Biochemical flare during postpartum in PBC and PSC.
