# Supplementary material for: Cholestatic Liver Disease and Pregnancy: A Systematic Review and Meta-Analysis
Source: J Clin Med. 2022 Feb 18;11(4):1068. doi: 10.3390/jcm11041068 (PMC8875982; doi:10.3390/jcm11041068)
Supplement: Supplementary file 1 [file jcm-11-01068-s001.zip › jcm-1585680-supplementary.pdf]

Supplementary Material

Table S1. Search strategy

**PubMed**
("Liver cirrhosis, Biliary"[Mesh] OR "biliary liver cirrhosis"[tiab] OR "primary biliary cholangitis"[tiab] OR "primary biliary cirrhosis"[tiab] OR "cholestatic liver disease"[tiab] OR "PBC"[tiab] OR "Cholangitis, Sclerosing"[Mesh] OR "Primary sclerosing cholangitis"[tw] OR "PSC"[tiab])<br>AND<br>("Pregnancy"[MeSH] OR Pregnancy[tw] OR pregnant[tw] OR "Pregnant Women"[MeSH] OR "Pregnant Women"[tw] OR "Pregnant Woman"[tw] OR gestation[tw] OR "gravidity"[MeSH] OR gravidity[tw])<br>AND<br>("Pregnancy Outcome"[MeSH] OR "pregnancy outcome*"[tiab] OR "Pregnancy Complications"[MeSH] OR "pregnancy complication*"[tiab] OR "Treatment Outcome"[MeSH] OR "treatment outcome*"[tiab] OR "infant, newborn"[MeSH] OR "newborn"[tiab] or "infant"[tiab])
**EMBASE**
"biliary cirrhosis"/exp OR "biliary cirrhosis" OR "primary biliary cirrhosis"/exp OR "primary biliary cirrhosis" OR "cholestatic liver disease"/exp OR "cholestatic liver disease" OR "sclerosing cholangitis"/exp OR "sclerosing cholangitis"<br>AND<br>"pregnancy"/exp OR "pregnancy" OR "pregnant woman"/exp OR "pregnant woman"
**Web of Science**
TS=("biliary liver cirrhosis" OR "primary biliary cholangitis" OR "primary biliary cirrhosis" OR "cholestatic liver disease" OR "PBC" OR "sclerosing cholangitis" OR "primary sclerosing cholangitis" OR "PSC")<br>AND<br>TS=("pregnancy" OR "pregnant" OR "pregnant women" OR "pregnant woman" OR "gestation" OR "gravidity")
**Cochrane Reviews**
MeSH descriptor: [Liver Cirrhosis, Biliary] explode all trees OR MeSH descriptor: [Cholangitis, Sclerosing] explode all trees OR "biliary liver cirrhosis" OR "primary biliary cholangitis" OR "primary biliary cirrhosis" OR "cholestatic liver disease" OR PBC OR "primary sclerosing cholangitis" OR PSC<br>AND<br>MeSH descriptor: [Pregnancy] explode all trees OR MeSH descriptor: [Pregnant Women] explode all trees OR MeSH descriptor: [Gravidity] explode all trees OR pregnancy OR pregnant OR "pregnant women" OR "pregnant woman" OR gestation OR gravidity

Table S2. Diagnostic criteria of Primary Biliary cholangitis and Primary Sclerosing Cholangitis based on AASL and EASL guidelines

**PBC**	
AASLD guidelines	A diagnosis of PBC per AASLD guidelines is established when two of the following three findings are present:<br>(1) Elevated serum alkaline phosphatase (ALP),<br>(2) Presence of antimitochondrial antibody (AMA) or other autoantibodies (anti-sp100 or anti-gp210)<br>(3) If AMA is negative, then subsequent liver biopsy showing nonsuppurative biliary ductal destruction.
EASL guidelines	PBC equates to at least two of the following:<br>(1) ALP ≥2x upper limit of normal (ULN) or GGT >5x ULN;<br>(2) AMA titer >1:40;<br>(3) Florid bile duct lesion on histology.
**PSC**	
AASLD guidelines	A diagnosis of PSC is made when patients with abnormal cholestatic liver tests have:<br>• Multifocal strictures in the intra-, and extra-hepatic bile ducts, as well as segmental dilations on cholangiography (magnetic resonance cholangiography, endoscopic retrograde cholangiography, or percutaneous cholangiography), and secondary causes of sclerosing cholangitis have been ruled out.<br>• Histological confirmation is needed in patients with normal cholangiograms.
EASL guidelines	A diagnosis of PSC is made in patients with<br>• Elevated serum markers of cholestasis not otherwise explained,<br>• When magnetic resonance cholangiopancreatography (MRCP) or endoscopic cholangiopancreatography (ERCP) show characteristic bile duct changes with multifocal strictures and segmental dilatations,<br>• And causes of secondary sclerosing cholangitis and other cholestatic disorders are excluded.

PBC—Primary biliary cholangitis; PSC—Primary sclerosing cholangitis; AASLD— American Association for the Study of Liver Diseases; EASL— European Association for the Study of the Liver.

Table S3. Excluded studies

Study	Reason for exclusion
Matsumori, A.; Yoneda, S.; Kojima, H.; An, S.; Uemura, M.; Fukui, H.; Yamane, Y.; Nishimura, K.; Yoshikawa, M. A Case of Primary Biliary Cirrhosis Generated during Pregnancy. J. Nara Med. Assoc. 2003, 54 (4), 257–262.	Case Report <3 Patients
Mizuno, M.; Kohda, H.; Kanai, M.; Murazumi, K.; Ohta, H.; Uehara, S.; Ishikawa, Y.; Hasebe, C.; Ono, M.; et al. A CASE OF PRIMARY BILIARY CIRRHOSIS WHICH DEVELOPED AFTER THIRD DELIVERY. Gastroenterol. Endosc. 1988, 30 (11), 2652–2658.	Case Report <3 Patients<br>Article Is in Japanese
Mizuno, S.; Ueno, T.; Simasaki, H.; Yasuhara, S.; Takeda, R.; Nakanuma, Y. [A Case of Primary Biliary Cirrhosis, Manifested by Pregnancy]. Nihon Shokakibyo Gakkai Zasshi 1985, 82 (10), 2642–2646.	Case Report <3 Patients<br>Article Is in Japanese
Siva, S.; Muchero, R.; Nangrahary, M.; Senaratne, S. An Unusual Case of Liver Disease in Pregnancy. Aust. N. Z. J. Obstet. Gynaecol. 2018, 58, 76. https://doi.org/10.1111/ajo.12874.	Case Report <3 Patients
Parikh-Patel, A.; Gold, E. B.; Utts, J. M.; Gershwin, M. E. Association between Parity and Primary Biliary Cirrhosis. Am. J. Epidemiol. 2001, 153 (11), S138–S138.	Wrong Outcomes: does not specify if pregnancy occurred
Varma, R. R. Course and Prognosis of Pregnancy in Women with Liver Disease. Semin Liver Dis 1987, 7 (1), 59–66. https://doi.org/10.1055/s-2008-1040565.	Review Article
Bouldouyre, M. A.; Dauphin, H.; Cherradou, N.; Gros, H. [Establishment and One-Year Evaluation of an Internal Medicine Consultation in a Maternity]. Sante Publique (Bucur.) 2018, 30 (5), 671–677. https://doi.org/10.3917/spub.186.0671.	Article in French
Frise, C. J.; Williamson, C. Gastrointestinal and Liver Disease in Pregnancy. Clin. Med. J. R. Coll. Physicians Lond. 2013, 13 (3), 269–274. https://doi.org/10.7861/clinmedicine.13-3-269.	Review Article
Matsubara, S.; Isoda, N.; Taniguchi, N. Jaundice as the First Manifestation of Primary Biliary Cirrhosis during Pregnancy: Measurement of Portal Vein Blood Flow. J Obstet Gynaecol Res 2011, 37 (7), 963–964. https://doi.org/10.1111/j.1447-0756.2011.01645.x	Case Report <3 Patients<br>Duplicate
Matsubara, S.; Isoda, N.; Taniguchi, N. Jaundice as the First Manifestation of Primary Biliary Cirrhosis during Pregnancy: Measurement of Portal Vein Blood Flow. J Obstet Gynaecol Res 2011, 37 (7), 963–964. https://doi.org/10.1111/j.1447-0756.2011.01645.x.	Case Report <3 Patients<br>Duplicate
Mincis, M. Liver Diseases in Pregnancy. Rev. Bras. Med. 2004, 61 (11), 695–702.	Review Article<br>Article in Portuguese
Nabhan, S.; Riely, C. A. Liver Diseases in Pregnancy. Article Seven in the Series. Pract. Gastroenterol. 1996, 20 (7), 14–37.	Review Article
Nir, A.; Sorokin, Y.; Abramovici, H.; Theodor, E. Pregnancy and Primary Biliary Cirrhosis. Int J Gynaecol Obstet 1989, 28 (3), 279–282. https://doi.org/10.1016/0020-7292(89)90731-5.	Case Report <3 Patients
Gossard, A. A.; Lindor, K. D. Pregnancy in a Patient with Primary Sclerosing Cholangitis. J Clin Gastroenterol 2002, 35 (4), 353–355. https://doi.org/10.1097/00004836-200210000-00014.	Case Report <3 Patients
Hedri, A.; Dionysopoulou, A.; Lindner, C.; Macchiella, D.; Steetskamp, J.; Hasenburg, A. Pregnancy in a Patient with Primary Sclerosing Cholangitis. Geburtshilfe Frauenheilkd. 2018, 78 (10). https://doi.org/10.1055/s-0038-1671541.	Case Report <3 Patients
Borhanmanesh, F.; Haghighi, P. Pregnancy in Patients with Cirrhosis of the Liver. Obstet Gynecol 1970, 36 (2), 315–324.	Wrong patient population
Wong, K. K.; Goh, K. L. Pregnancy in Primary Biliary Cirrhosis. Eur J Obstet Gynecol Reprod Biol 1992, 45 (2), 149–151. https://doi.org/10.1016/0028-2243(92)90232-n.	Case Report <3 Patients

Goh, S. K.; Gull, S. E.; Alexander, G. J. Pregnancy in Primary Biliary Cirrhosis Complicated by Portal Hypertension: Report of a Case and Review of the Literature. Bjog 2001, 108 (7), 760–762. https://doi.org/10.1111/j.1471-0528.2001.00189.x.	Case Report <3 Patients
Ozdemir, O.; Karaahmet, F.; Sari, E.; Yakut, K.; Ertugrul, F. A.; Atalay, C. Preterm Birth Related to Post-Endoscopic Retrograde Cholangiopancreatography Pancreatitis in Pregnancy with Newly Diagnosed Primary Sclerosing Cholangitis. J Obstet Gynaecol 2015, 35 (3), 305–306. https://doi.org/10.3109/01443615.2014.948411.	Case Report <3 Patients
Lopes, I. F.; Palma dos Reis, C. R.; Alves, M. J.; Calinas, F.; Borges, M. A. Primary Biliary Cholangitis: A Rare Diagnosis during Pregnancy. Obstet. Med. 2021. https://doi.org/10.1177/1753495X211008290.	Case Report <3 Patients
Marpeau, L.; Benifla, J. L.; Chazoulliere, O.; Larue, L.; Pigne, A.; Poupon, R.; Barrat, J. Primary Biliary Cirrhosis (BPC) and Pregnancy. A Case Report. Review of the Literature. J. Gynecol. Obstet. Biol. Reprod. (Paris) 1991, 20 (6), 805–807.	Case Report <3 Patients Article in French
Herijgers, P.; Van Coppenolle, L. Primary Biliary Cirrhosis and Pregnancy. Tijdschr. Voor Geneeskd. 1991, 47 (5), 353–357.	Foreign language without ability to interpret
Mantha, U.; Tebbutt, H. Primary Biliary Cirrhosis and Pregnancy. J. Obstet. Gynaecol. 1993, 13 (3), 177–178.	Case Report <3 Patients
Pajares Villarroya, R.; Castillo Grau, P.; Manceñido Marcos, N.; Navajas León, F. J.; Hervías Cruz, D.; Erdozain Sosa, J. C.; Segura Cabral, J. M. Primary Biliary Cirrhosis and Pregnancy: Benefit of Ursodeoxycholic Acid Therapy [2]. Gastroenterol Hepatol 2003, 26 (9), 615. https://doi.org/10.1157/13054454.	Foreign Language without Ability to Interpret
Pajares Villarroya, R.; Castillo Grau, P.; Manceñido Marcos, N.; Navajas León, F. J.; Hervías Cruz, D.; Erdozain Sosa, J. C.; Segura Cabral, J. M. [Primary Biliary Cirrhosis and Pregnancy: Benefit of Ursodeoxycholic Acid Therapy]. Gastroenterol Hepatol 2003, 26 (9), 615. https://doi.org/10.1016/s0210-5705(03)70416-6.	Duplicate
Marpeau, L.; Benifla, J. L.; Chazoullière, O.; Larue, L.; Pigné, A.; Poupon, R.; Barrat, J. [Primary Biliary Cirrhosis and Pregnancy. Apropos of a Clinical Case. Review of the Literature]. J Gynecol Obstet Biol Reprod Paris 1991, 20 (6), 805–807	Case Report <3 Patients
Ducarme, G.; Bernuau, J.; Luton, D. [Primary Biliary Cirrhosis and Pregnancy]. J Gynecol Obstet Biol Reprod Paris 2014, 43 (5), 335–341. https://doi.org/10.1016/j.jgyn.2013.03.016.	Review Article
Rabinovitz, M.; Appasamy, R.; Finkelstein, S. Primary Biliary Cirrhosis Diagnosed during Pregnancy. Does It Have a Different Outcome? Dig Sci 1995, 40 (3), 571–574. https://doi.org/10.1007/bf02064371.	Case Report <3 Patients
Belloni, G.; Talamazzini, A.; Soldati, P. M.; Bernini, L.; Di Gennaro, F.; Bergamaschi, P. Primary Biliary Cirrhosis Risen in Pregnancy: A Case Report. Med. - Riv. Della Encicl. Medica Ital. 1990, 10 (1), 31–32.	Case Report <3 Patients Article in Italian
Belloni, G.; Talamazzini, A.; Soldati, P. M.; Bernini, L.; Di Gennaro, F.; Bergamaschi, P. [Primary Biliary Cirrhosis with Onset in Pregnancy: A Case Report]. Med. Firenze 1990, 10 (1), 31–32.	Duplicate
Kammeijer, C. Q.; De Man, R. A.; De Groot, C. J. Primary Sclerosing Cholangitis and Pregnancy. Clin Pr. 2011, 1 (3), e55. https://doi.org/10.4081/cp.2011.e55.	Review Article
Landon, M. B.; Soloway, R. D.; Freedman, L. J.; Gabbe, S. G. Primary Sclerosing Cholangitis and Pregnancy. Obstet Gynecol 1987, 69 (3 Pt 2), 457–460.	Case Report <3 Patients
Leftwich, H.; Fang, Y. M. V.; Borgida, A.; Crombleholme, W. Primary Sclerosing Cholangitis in Pregnancy Refractory to Ursodeoxycholic Acid Treatment. J. Reprod. Med. Obstet. Gynecol. 2010, 55 (11–12), 517–519.	Case Report <3 Patients
Leftwich, H.; Fang, Y. M.; Borgida, A.; Crombleholme, W. Primary Sclerosing Cholangitis in Pregnancy Refractory to Ursodeoxycholic Acid Treatment: A Case Report. J Reprod Med 2010, 55 (11–12), 517–519.	
Christensen, K. L.; Andersen, B. N.; Vilstrup, H. [Primary Sclerosing Cholangitis with Itching Treated during Pregnancy with Ursodeoxycholic Acid]. Ugeskr Laeger 1997, 159 (48), 7151–7153.	Case Report <3 Patients Article in Danish
Christensen, K. L.; Andersen, B. N.; Vilstrup, H. Primary Sclerosing Cholangitis with Pruritis Treated during Pregnancy with Ursodeoxycholic Acid. Ugeskr Laeger 1997, 159 (48), 7151–7153.	Duplicate
Sujana Kumar, V.; Qumosani, K.; Taylor, T.; Sun, D. Primary Sclerosing Cholangitis: A New Case of Cirrhosis in Pregnancy. Obstet. Med. 2020. https://doi.org/10.1177/1753495X20972828.	Case Report <3 Patients

Slade, L.; McKendrick, L.; Grivell, R. Primary Sclerosing Cholangitis: A Rare Cause of Liver Dysfunction in Pregnancy. Obstet. Med. 2021. https://doi.org/10.1177/1753495X21991406.	Case Report <3 Patients
Ponce De Leon Hector, H.; Rodolfo, B.; Castelleto Roberto Argento Amalia-V, M.; Chopita Nestor, F. A. N. RELAPSING CHOLESTASIS OF PREGNANCY AND PRIMARY BILIARY CIRRHOSIS NONSUPPURATIVE DESTRUCTIVE CHRONIC CHOLANGITIS. Acta Gastroenterol Latinoam 1983, 13 (2), 210–211.	Foreign language without ability to interpret
Faulkes, R. E.; Chauhan, A.; Knox, E.; Johnston, T.; Thompson, F.; Ferguson, J. Review Article: Chronic Liver Disease and Pregnancy. Aliment. Pharmacol. Ther. 2020, 52 (3), 420–429. https://doi.org/10.1111/apt.15908.	Review Article
Alallam, A.; Barth, D.; Heathcote, E. J. Role of Plasmapheresis in the Treatment of Severe Pruritus in Pregnant Patients with Primary Biliary Cirrhosis: Case Reports. Can J Gastroenterol 2008, 22 (5), 505–507. https://doi.org/10.1155/2008/969826.	Case Report <3 Patients
Parikh-Patel, A.; Gold, E.; Utts, J.; Gershwin, M. E. The Association between Gravidity and Primary Biliary Cirrhosis. Ann Epidemiol 2002, 12 (4), 264–272. https://doi.org/10.1016/s1047-2797(01)00277-0.	Wrong Outcomes
Rudi, J.; Schönig, T.; Stremmel, W. Treatment with Ursodeoxycholic Acid in Primary Biliary Cirrhosis during Pregnancy. Z Gastroenterol 1996, 34 (3), 188–191.	Foreign language without ability to interpret
Holtmeier, J.; Leuschner, M.; Holtmeier, W.; Stiehl, A.; Klein, R.; Leuschner, U. Ursodeoxycholic Acid in the Treatment of Primary Biliary Cirrhosis (PBC) and Primary Sclerosing Cholangitis (PSC) in Pregnancy. Hepatology 2002, 36 (4), 491A-491A.	Wrong Outcomes
Holtmeier, J.; Leuschner, M.; Stiehl, A.; Klein, R.; Leuschner, U.; Leuschner, U.; Berg, P. A.; Holtmeier, J. Ursodeoxycholic Acid in the Treatment of Primary Biliary Cirrhosis and Primary Sclerosing Cholangitis in Pregnancy; 2002; Vol. 129A, p 74.	Review Article
Sun, Y.; Haapanen, K.; Li, B.; Zhang, W.; Van De Water, J.; Gershwin, M. E. Women and Primary Biliary Cirrhosis. Clin. Rev. Allergy Immunol. 2014. https://doi.org/10.1007/s12016-014-8449-4.	Review Article;
Sun, Y.; Haapanen, K.; Li, B.; Zhang, W.; Van de Water, J.; Gershwin, M. E. Women and Primary Biliary Cirrhosis.Clin Rev Allergy Immunol 2015, 48 (2–3), 285–300. https://doi.org/10.1007/s12016-014-8449-4.	Review Article

Table S4. Assessment of quality of a Cohort study – Newcastle Ottawa Scale

**Selection** (tick one box in each section)		
1. Representativeness of the intervention cohort		
a) Truly representative of the average, pregnant with cholestatic liver disease ★		☐
b) Somewhat representative of the pregnant with cholestatic liver disease ★		☐
c) Selected group of patients, e.g. only certain socio-economic groups/areas		☐
d) No description of the derivation of the cohort		☐
2. Selection of the non-intervention cohort		
a) Drawn from the same community as the intervention cohort	★	☐
b) Drawn from a different source		☐
c) No description of the derivation of the non-intervention cohort		☐
3. Ascertainment of intervention		
a) Secure record (eg health care record)	★	☐
b) Structured interview	★	☐
c) Written self-report		☐
d) Other / no description		☐
4. Demonstration that outcome of interest was not present at start of study		
a) Yes	★	☐
b) No		☐

**Comparability (**tick one or both boxes, as appropriate)	
1. Comparability of cohorts on the basis of the design or analysis<br>a) Most important factors of adjustment ★<br>b) Study controls for any additional factors ★	☐<br>☐
**Outcome** (tick one box in each section)	
1. Assessment of outcome<br>a) Independent blind assessment ★<br>b) Record linkage ★<br>c) Self-report<br>d) Other / no description	☐<br>☐<br>☐<br>☐
2. Was follow up long enough for outcomes to occur<br>a) Yes ★<br>b) No	☐<br>☐
3. Adequacy of follow up of cohorts<br>a) Complete follow up: all subjects accounted for ★<br>b) Subjects lost to follow up unlikely to introduce bias: number lost <= 20%, or description of those lost suggesting no different from those followed ★<br>c) Follow up rate < 80% (select an adequate %) and no description of those lost<br>d) No statement	☐<br>☐<br>☐<br>☐

Table S5. Assessment of quality of a Case-Control study – Newcastle Ottawa Scale

**Selection** (tick one box in each section)	
1. is the case definition adequate?<br>a) Yes, with independent validation ★<br>b) Yes, e.g., record linkage or based on self-report<br>c) No description	☐<br>☐<br>☐
2. Representativeness of the cases<br>a) Consecutive or obviously representative series of cases ★<br>b) Potential for selection biases or not stated	☐<br>☐
3. Selection of controls:<br>a) Controls were selected from the same source population as the cases ★<br>b) Controls were selected from a different source population<br>c) No description	☐<br>☐<br>☐
4. Definition of controls:<br>a) No history of disease (Cholestatic liver disease) ★<br>b) No description of source	☐<br>☐
**Comparability** (tick one or both boxes, as appropriate)	
1. Comparability of cases and controls on the basis of the design or analysis controlled for confounders:<br>a) The study controls for age ★<br>b) Study controls for any additional factors (e.g. socio-economic status, education) ★<br>c) Cohorts are not comparable on the basis of the design or analysis controlled for confounders	☐<br>☐<br>☐
**Exposure** (tick one box in each section)	

1. Assessment of exposure<br><br>a) Secure record ★<br>b) Structured interview where blind to case/control status ★<br>c) Interview not blinded to case/control status<br>d) Written self-report or medical record only<br>e) No description	☐<br>☐<br>☐<br>☐<br>☐
2. Same method of ascertainment for cases and controls:<br><br>a) Yes ★<br>b) No	☐<br>☐
3. Non-response rate:<br><br>a) Same rate for both groups ★<br>b) Non-respondents described<br>c) Rate different between cases and controls with no description	☐<br>☐<br>☐

Table S6. Quality Appraisal Checklist for Case Series Studies[1]

**Study objective**		
**1.**	**Was the hypothesis/aim/objective of the study clearly stated?**	Yes ☐<br>Partial ☐<br>No ☐
**Study design**		
**2.**	**Was the study conducted prospectively?**	Yes ☐<br>Unclear ☐<br>No ☐
**3.**	**Were the cases collected in more than one centre?**	Yes ☐<br>Unclear ☐<br>No ☐
**4.**	**Were patients recruited consecutively?**	Yes ☐<br>Unclear ☐<br>No ☐
**Study population**		
**5.**	**Were the characteristics of the patients included in the study described?**	Yes ☐<br>Partial ☐<br>No ☐
**6.**	**Were the eligibility criteria (i.e. inclusion and exclusion criteria) for entry into the study clearly stated?**	Yes ☐<br>Partial ☐<br>No ☐
**7.**	**Did patients enter the study at a similar point in the disease?**	Yes ☐<br>Unclear ☐<br>No ☐
**Study population**		
**8.**	**Were relevant outcome measures established a priori?**	Yes ☐

		Unclear ☐<br>No ☐
9.	**Were outcome assessors blinded to the intervention that patients received?**	Yes ☐<br>Unclear ☐<br>No ☐
10.	**Were the relevant outcomes measured using appropriate objective/subjective methods?**	Yes ☐<br>Partial ☐<br>No ☐
11.	**Were the relevant outcome measures made before, during pregnancy and in the post-partum period?**	Yes ☐<br>Unclear ☐<br>No ☐
**Statistical analysis**		
12.	**Were the statistical tests used to assess the relevant outcomes appropriate?**	Yes ☐<br>Unclear ☐<br>No ☐
**Results and conclusions**		
13.	**Was follow-up long enough for important events and outcomes to occur?**	Yes ☐<br>Unclear ☐<br>No ☐
14.	**Were losses to follow-up reported?**	Yes ☐<br>Unclear ☐<br>No ☐
15.	**Did the study provided estimates of random variability in the data analysis of relevant outcomes?**	Yes ☐<br>Partial ☐<br>No ☐
16.	**Were the conclusions of the study supported by results?**	Yes ☐<br>Partial ☐<br>No ☐
**Competing interests and sources of support**		
17.	**Were both competing interests and sources of support for the study reported?**	Yes ☐<br>Partial ☐<br>No ☐

[1] This checklist was adapted from the Institute of Health Economics (IHE) Quality Appraisal of Case Series Studies Checklist. Available from: http://www.ihe.ca/research-programs/rmd/cssqac/cssqac-about

Table S7. GRADE assessment for fetal outcomes in PSC pregnancies

Certainty assessment						№ of patients		Effect		Certainty[2]	Importance
**№ of studies**	**Study design**	**Risk of bias**	**Inconsistency**	**Imprecision**	**Other considerations**	**PSC**	**Control**	**Relative (95% CI)**	**Absolute (95% CI)**		
**Preterm birth (assessed with: OR)**											
2	Observational	not serious	not serious	serious[1]	strong association	45/263 (17.1%)	11327/2189 4568 (0.1%)	**OR 3.69** (2.65 to 5.12)	**1 more per 1,000** (from 1 more to 2 more)	⊕⊕◯◯ Low	IMPORTANT
**Birth defects (assessed with: OR)**											
2	Observational	not serious	not serious	serious[1]	strong association	31/453 (6.8%)	82853/2214 649 (3.7%)	**OR 2.25** (0.81 to 6.25)	**43 more per 1,000** (from 7 fewer to 158 more)	⊕⊕◯◯ Low	IMPORTANT

PSC—Primary sclerosing cholangitis; CI—Confidence Interval; OR—Odds ratio

[1] The effect estimate comes from only two studies with few events.

[2] GRADE definition:

High -Further research is very unlikely to change our confidence in the estimate of effect.

Moderate - Further research is likely to have an important impact on our confidence in the estimate of effect and may change the estimate.

Low - Further research is very likely to have an important impact on our confidence in the estimate of effect and is likely to change the estimate.

Very low -Any estimate of effect is very uncertain.

Table S8. GRADE assessment for fetal outcomes in PBC pregnancies

Certainty assessment						№ of patients		Effect		Certainty[2]	Importance
**№ of studies**	**Study design**	**Risk of bias**	**Inconsistency**	**Imprecision**	**Other considerations**	**PBC**	**Control**	**Relative (95% CI)**	**Absolute (95% CI)**		
**Preterm birth (assessed with: OR)**											
2	Observational	not serious	not serious	serious[1]	strong association	9/35 (25.7%)	0.0%	**OR 3.95** (2.90 to 5.38)	**0 fewer per 1,000** (from 0 fewer to 0 fewer)	⊕⊕◯◯ Low	IMPORTANT

PBC—Primary biliary cholangitis; CI—Confidence Interval; OR—Odds ratio
[1] The effect estimate comes from only two small studies with few events.
[2] GRADE definition:
High -Further research is very unlikely to change our confidence in the estimate of effect.
Moderate - Further research is likely to have an important impact on our confidence in the estimate of effect and may change the estimate.
Low - Further research is very likely to have an important impact on our confidence in the estimate of effect and is likely to change the estimate.
Very low -Any estimate of effect is very uncertain.

Table S9. GRADE assessment for pruritus in pregnant women with PBC

Certainty assessment						№ of patients		Effect		Certainty[3]	Importance
**№ of studies**	**Study design**	**Risk of bias**	**Inconsistency**	**Imprecision**	**Other considerations**	**Group 1**	**Group 2**	**Relative (95% CI)**	**Absolute (95% CI)**		
**Pruritus during pregnancy vs before gestation (assessed with: OR)**						**Pruritus during pregnancy**	**Pruritus before gestation**				
3	Observational studies	not serious	not serious	serious[1]	strong association	39/62 (62.9%)	18/61 (29.5%)	**OR 4.35** (1.98 to 9.57)	**350 more per 1,000** (From 158 more to 505 more)	⊕⊕◯◯ Low	IMPORTANT
**Post-partum pruritus vs pruritus during pregnancy (assessed with: OR)**						**Post-partum pruritus**	**Pruritus during pregnancy**				
4	Observational studies	not serious	not serious	serious[2]	none	21/71 (29.6%)	39/71 (54.9%)	**OR 0.36** (0.09 to 1.38)	**244 fewer per 1,000** (From 450 fewer to 78 more)	⊕◯◯◯ Very low	IMPORTANT

PBC—Primary biliary cholangitis; CI—Confidence Interval; OR—Odds ratio
[1] The effect estimate comes from only three small studies with few events.
[2] The effect estimate comes from four small studies with few events.
[3] GRADE definition:
High -Further research is very unlikely to change our confidence in the estimate of effect.
Moderate - Further research is likely to have an important impact on our confidence in the estimate of effect and may change the estimate.
Low - Further research is very likely to have an important impact on our confidence in the estimate of effect and is likely to change the estimate.
Very low -Any estimate of effect is very uncertain.

Table S10. GRADE assessment for pruritus in pregnant women with PSC

Certainty assessment						№ of patients		Effect		Certainty[2]	Importance
№ of studies	Study design	Risk of bias	Inconsistency	Imprecision	Other considerations	Group 1	Group 2	Relative (95% CI)	Absolute (95% CI)		
**Pruritus during pregnancy vs before gestation (assessed with: OR)**						**Pruritus during pregnancy**	**Pruritus before gestation**				
3	Observational studies	not serious	not serious	serious[1]	strong association	28/80 (35.0%)	14/80 (17.5%)	**OR 2.51** (1.20 to 5.27)	**172 more per 1,000** (From 28 more to 353 more)	⊕⊕◯◯ Low	IMPORTANT
**Post-partum pruritus vs pruritus during pregnancy (assessed with: OR)**						**Post-partum pruritus**	**Pruritus during pregnancy**				
3	Observational studies	not serious	not serious	serious[1]	none	16/80 (20.0%)	28/80 (35.0%)	**OR 0.47** (0.23 to 0.96)	**148 fewer per 1,000** (From 240 fewer to 9 fewer)	⊕◯◯◯ Very low	IMPORTANT

PSC—Primary sclerosing cholangitis; CI—Confidence Interval; OR—Odds ratio

[1] The effect estimate comes from only three small studies with few events.

[2] GRADE definition:

High -Further research is very unlikely to change our confidence in the estimate of effect.

Moderate - Further research is likely to have an important impact on our confidence in the estimate of effect and may change the estimate.

Low - Further research is very likely to have an important impact on our confidence in the estimate of effect and is likely to change the estimate.

Very low -Any estimate of effect is very uncertain.

Table S11. GRADE assessment for Biochemical flare during postpartum in PBC and PSC

Certainty assessment						№ of patients		Effect		Certainty[3]	Importance
№ of studies	Study design	Risk of bias	Inconsistency	Imprecision	Other considerations	Post-partum	During pregnancy	Relative (95% CI)	Absolute (95% CI)		
**Biochemical flare in PBC patients (assessed with: OR)**											

3	Observational	not serious	not serious	serious[1]	none	38/62 (61.3%)	16/53 (30.2%)	**OR 2.00** (1.27 to 3.13)	**162 more per 1,000** (from 53 more to 273 more)	⨁◯◯◯ Very low	IMPORTANT
**Biochemical flare in PSC patients (assessed with: OR)**											
2	Observational	not serious	not serious	serious[2]	none	14/38 (36.8%)	7/38 (18.4%)	**OR 1.99** (1.34 to 2.94)	**126 more per 1,000** (from 48 more to 215 more)	⨁◯◯◯ Very low	IMPORTANT

PBC—Primary biliary cholangitis; PSC—Primary sclerosing cholangitis; CI—Confidence Interval; OR—Odds ratio

[1] The effect estimate comes from only three small studies with few events.

[2] The effect estimate comes from only two small studies with few events.

[3] GRADE definition:

High -Further research is very unlikely to change our confidence in the estimate of effect.

Moderate - Further research is likely to have an important impact on our confidence in the estimate of effect and may change the estimate.

Low - Further research is very likely to have an important impact on our confidence in the estimate of effect and is likely to change the estimate.

Very low -Any estimate of effect is very uncertain.
